# Long-term Use of Clozapine is Protective for Bone Density in Patients with Schizophrenia

**DOI:** 10.1038/s41598-019-40691-7

**Published:** 2019-03-07

**Authors:** Chieh-Hsin Lin, Chun-Yuan Lin, Hong-Song Wang, Hsien-Yuan Lane

**Affiliations:** 1 Department of Psychiatry, Kaohsiung Chang Gung Memorial Hospital, Chang Gung University College of Medicine, Kaohsiung, Taiwan; 2 Graduate Institute of Biomedical Sciences, China Medical University, Taichung, Taiwan; 3 School of Medicine, Chang Gung University, Taoyuan, Taiwan; 4 Tsaotun Psychiatric Center, Ministry of Health and Welfare, Nantou, Taiwan; 5 School of Medicine, Chung Shan Medical University, Taichung, Taiwan; 6 National Changhua University of Education, Changhua, Taiwan; 7 Psychiatric department, Changhua Hospital, Ministry of Health & Welfare, Changhua, Taiwan; 8 Department of Psychiatry & Brain Disease Research Center, China Medical University Hospital, Taichung, Taiwan; 9 Department of Psychology, College of Medical and Health Sciences, Asia University, Taichung, Taiwan

## Abstract

Low bone mineral density (BMD) prevails among patients with schizophrenia. Antipsychotics use plays an important role in BMD. Previous cross-section study suggests that clozapine treatment may benefit BMD of women with schizophrenia. However, the effect of long-term clozapine therapy on BMD remains unknown. This prospective study compared clozapine and non-clozapine antipsychotics in long-term effects on BMD among both men and women with schizophrenia. Patients with schizophrenia and age-matched healthy individuals were enrolled from two centers. All patients, including clozapine receivers and non-clozapine antipsychotics recipients, kept clinically stable with unchanged antipsychotics and doses for at least 6 months at enrollment and during the follow-up period. BMD was examined by dual-energy X-ray absorptiometer upon enrollment and at 1- or 3-year follow-up. Thorough clinical and laboratory variables were measured too. The mean BMD of patients receiving clozapine was higher than that of the non-clozapine patients at both enrollment and follow-up. Overall, the patients in the clozapine group gained BMD, while those in the non-clozapine group lost BMD after 1–3 years (p = 0.015). There was no significant difference of BMD change between clozapine-treated patients and healthy controls. Factors associated with BMD change in the clozapine group included calcium level (B = −0.607, *p* = 0.021) and T3 level (B = −0.077, *p* = 0.007). This longitudinal study suggests that long-term clozapine treatment may protect BMD compared to prolactin-raising and non-clozapine prolactin-sparing antipsychotics among patients with schizophrenia. Future prospective studies are warranted to testify whether switching from non-clozapine antipsychotics to clozapine can rescue BMD.

## Introduction

Decreased bone mineral density (BMD), partially due to medication use, is widespread among both male and female patients with schizophrenia^1,2^. Low BMD, associated with osteoporosis and fracture, contributes to high morbidity and mortality^3,4^. Moreover, patients with schizophrenia, while receiving antipsychotic treatment, are prone to fall, further worsening their life quality^5^.

The long-term impacts of various antipsychotics on BMD have been gaining more attention^6,7^. However, previous studies showed inconsistent findings. Abraham *et al*. did not find a significant BMD change in a 12-month prospective study but noticed higher rates of bone formation and resorption in patients with high prolactin levels^8^. Among antipsychotics, prolactin-raising (PR) antipsychotics, are more likely to influence BMD of patients, due to antipsychotic-induced hyperprolactinemia and/or secondary hypogonadism^9,10^. In accordance, Meaney and O’Keane found BMD loss in patients receiving PR antipsychotics but BMD gain in the prolactin-sparing (PS) recipients over one year^11^. While the effect of hyperprolactinemia stays inconclusive^8,12,13^, a longer observational study, with average follow-up at 3.4 ± 1.6 (SD) years, revealed a negative influence of PR antipsychotics on BMD^14^. Different PS antipsychotics generate diverse short-term impacts on BMD^15^. Of note, the long-term effects of different PS antipsychotics on BMD remain unknown.

Our previous study found that women with schizophrenia receiving clozapine had better BMD than those taking PR antipsychotic treatment; however, there was no correlation between prolactin level and BMD^12^. Cui *et al*. also found that schizophrenia patients receiving clozapine had higher BMD than those receiving non-clozapine^16^. Patients with refractory schizophrenia who receive clozapine therapy usually need long duration of treatment, because it is the last-line therapy for schizophrenia even while novel compounds are under development^17,18^. Therefore, long-term clozapine effects on BMD require elucidation. Animal study suggests that clozapine can interact with the glycine site of the N-methyl-D-aspartic acid receptor (NMDAR)^19^ and chronic clozapine administration can up-regulate NMDARs^20,21^. NMDARs are expressed in osteoblasts and osteoclasts^22,23^, and down-regulation of NMDARs may result in the decrease of osteogenesis^24^. It has not yet been clear whether clozapine exerts different effects on BMD compared to other antipsychotics, particularly non-clozapine PS antipsychotics that do not cause hyperprolactinemia. This study prospectively followed up BMD for one to three years in both male and female patients with schizophrenia under stable antipsychotics treatment and in healthy men and women, aiming to investigate the long-term effect of clozapine vs. non-clozapine antipsychotics (including PR and non-clozapine PS antipsychotics) on BMD.

## Results

### Subjects

A total of 155 participants (111 schizophrenia patients and 44 healthy controls) were recruited. Among 111 patients with schizophrenia, 22 patients (19.8%) were from the outpatient clinics and the other 89 (80.2%) were from the inpatient units. Patients with schizophrenia were classified into two groups accordingly to the antipsychotics they had been taking: clozapine group and non-clozapine antipsychotics group. Overall, there were three groups, including two groups of schizophrenia patients and one group of healthy individuals.

The mean age was similar among the three groups. The percentage of women in healthy individuals was higher than those in schizophrenia groups (p = 0.004, χ^2^ test). The healthy individuals had higher education level, lower body weight and BMI, smaller waist and hip circumstances than the schizophrenia patients (all p values < 0.001, Table 1). Between the two groups of schizophrenia patients, patients in the clozapine group had lower antipsychotic dose and severity of psychotic symptoms (mainly in the negative and general-subscales of PANSS) (Table 1). The global functioning, duration of disease, duration of antipsychotic treatment, and concomitant mood stabilizers use were similar between the two patient groups (all p values > 0.05, Table 1). Healthy individuals had lower alkaline phosphatase level, higher estradiol level and lower testosterone level than patients with schizophrenia. Patients in the clozapine group had higher TSH level than non-clozapine group (p = 0.011, Mann-Whitney U test). Patients in the non-clozapine group had higher prolactin level and higher percentage of hyperprolactinemia than the clozapine group and healthy controls (both p values < 0.001). Patients in the clozapine group had higher baseline BMD (determined by DEXA T score and DEXA Z score) than patients in the non-clozapine group (p = 0.007 and 0.017, respectively, post-hoc test using Bonferroni method). Patients in the non-clozapine group had higher percentage of baseline LBMDZ than patients in the clozapine group and healthy controls (p = 0.017 and < 0.001, respectively, Fisher’s Exact test). The three groups had similar calcium, T3 and cortisol levels (all p values > 0.05).

**Table 1:** Demographic and clinical characteristics of patients with schizophrenia and healthy individuals.

	Controls (N = 44)	Non-clozapine antipsychotics (N = 69)	Clozapine (N = 42)	P value
Age, year, mean (SD)	40.2 (8.6)	41.8 (10.4)	41.1 (8.1)	0.682
Education, year, mean (SD)	15.5 (1.5)	10.6 (3.0)	11.3 (2.9)	<0.001^‡^
Body weight, Kg, mean (SD)	60.2 (11.3)	70.9 (12.0)	70.3 (11.7)	<0.001
Body mass index, mean (SD)	22.6 (3.0)	26.2 (4.2)	26.3 (3.9)	<0.001^‡^
Waist circumstance, cm, mean (SD)	71.3 (7.2)	94.6 (10.6)	94.6 (9.3)	<0.001^‡^
Hip circumstance, cm, mean (SD)	96.5 (2.8)	104.0 (8.8)	103.9 (8.2)	<0.001^‡^
Illness duration, month, mean (SD)	—	205.6 (111.3)	246.4 (95.9)	0.053^‡^
Duration of antipsychotic treatment, day mean (SD)	—	2832.3 (2518.0)	3435.6 (3134.4)	0.268^‡^
Chlorpromazine equivalence dose, mg/day, mean (SD)^※^	—	586.6 (454.5)	420.5 (173.8)	0.026^‡^
PANSS total score, mean (SD)	—	92.3 (14.4)	83.5 (14.5)	0.002
PANSS positive-subscale score	—	20.3 (4.8)	18.3 (3.9)	0.055
PANSS negative-subscale score	—	23.6 (3.6)	21.9 (4.4)	0.024
PANSS general-psychopathology score	—	48.5 (7.8)	43.4 (8.3)	0.002
Global Assessment of Functioning score, mean (SD)	—	41.9 (11.2)	44.4 (10.1)	0.096
Calcium, mg/dl, mean (SD)	9.1 (0.4)	9.0 (0.4)	9.0 (0.3)	0.409^‡^
Alkaline phosphatase, U/L, mean (SD)	50.6 (14.4)	66.0 (17.9)	69.4 (23.2)	<0.001^‡^
TSH, mIU/L, mean (SD)	1.7 (1.1)	1.5 (0.8)	2.9 (10.5)	0.011^‡^
T3, ng/dl, mean (SD)	94.5 (14.5)	96.9 (21.8)	98.8 (35.5)	0.846^‡^
Cortisol, μg/dl, mean (SD)	10.8 (4.6)	11.9 (4.1)	11.7 (4.4)	0.302^‡^
Estradiol, ng/ml, mean (SD)	83.9 (73.4)	38.4 (34.6)	45.4 (39.9)	<0.001^‡^
Testosterone, ng/ml, mean (SD)	1.5 (1.9)	2.5 (2.8)	2.6 (2.1)	0.015^‡^
Prolactin, ng/ml, mean (SD)	11.5 (7.0)	31.3 (26.9)	11.3 (7.6)	<0.001^‡^
Baseline DEXA T score, mean (SD)	0.0 (1.2)	−0.6 (1.1)	0.2 (1.5)	0.003
Baseline DEXA Z score, mean (SD)	0.3 (1.1)	−0.3 (1.1)	0.4 (1.5)	0.008
Gender, female, n (%)	31 (70.5)	29 (42.0)	17 (40.5)	0.004^†^
Concomitant all mood stabilizers^◎^	—	13 (18.8)	9 (21.4)	0.807^‡^
Concomitant lithium treatment	—	6 (8.7)	5 (11.9)	0.744^‡^
Concomitant valproate treatment	—	7 (10.1)	5 (11.9)	0.761^‡^
Concomitant carbamazepine treatment	—	0 (0.0)	1 (2.4)	0.375^‡^
Hyperprolactinemia, n (%)	4 (9.1)	40 (58.0)	4 (9.3)	<0.001^†^
Low bone mineral density using DEXA T score (LBMDT)§	8 (18.2)	25 (36.2)	8 (19.0)	0.054^†^
Low bone mineral density using DEXA Z score (LBMDZ)*	4 (9.1)	35 (50.7)	11 (26.2)	<0.001^†^

Abbreviations: PR, prolactin-raising; PS, prolactin-sparing; PANSS: Positive and Negative Syndrome Scale.

§LBMDT was defined as DEXA T score ≤ –1.

*LBMDZ was defined as DEXA Z score ≤ –1.

^◎^Mood stabilizers included valproate, carbamazepine and lithium.

^‡^Mann-Whitney U test, for variables with non-normal distributions.

^‡^Fisher’s exact test.

^†^Chi square test.

^※^Gardner DM, Murphy AL, O’Donnell H, Centorrino F, Baldessarini RJ International consensus study of antipsychotic dosing. The American journal of psychiatry 167: 686-93.

### Patients taking clozapine had more bone density gain than patients taking other antipsychotics

For overall subjects as whole (both subjects with 1-year follow-up and subjects with 3-year follow-up), healthy individuals gained more BMD than patients with schizophrenia (both clozapine and non-clozapine patients) after 1–3 years (DEXA Z scores differences 0.16 ± 0.47, −0.10 ± 0.66, respectively, p = 0.013, Mann-Whitney U test). Noteworthy, clozapine-treated patients and healthy controls gained similar BMD (p = 0.31, Mann-Whitney U test); on the contrary, non-clozapine antipsychotics patients significantly lost BMD, compared to healthy controls after 1–3 years (p = 0.003, Mann-Whitney U test) (Table 2).

**Table 2:** Bone mineral density change among patients with schizophrenia and healthy individuals.

	Controls	Non-clozapine antipsychotics	Clozapine	P value
**DEXA Z score, overall**	(N = 44)	(N = 69)	(N = 42)	
Baseline, mean (SD)	0.28 (1.09)	−0.28 (1.14)	0.38 (1.45)	0.008
Endpoint, mean (SD)	0.42 (1.08)	−0.44 (0.96)	0.41 (1.49)	<0.001^‡^
Difference, mean (SD)	0.16 (0.47)	−0.16 (0.66)	0.03 (0.64)	0.015^‡^
**DEXA Z score, 1 year follow up**		(N = 48)	(N = 29)	
Baseline, mean (SD)	—	−0.54 (1.10)	0.16 (1.49)	0.017
Endpoint, mean (SD)	—	−0.68 (0.88)	0.09 (1.47)	0.006
Difference, mean (SD)	—	−0.13 (0.56)	−0.08 (0.70)	0.808^‡^
**DEXA Z score, 3 year follow up**	(N = 44)	(N = 22)	(N = 13)	
Baseline, mean (SD)	0.28 (1.09)	0.28 (1.15)	0.88 (1.29)	0.228
Endpoint, mean (SD)	0.42 (1.08)	0.09 (0.93)	1.13 (1.29)	0.052^‡^
Difference, mean (SD)	0.16 (0.47)	−0.21 (0.87)	0.25 (0.41)	0.039^‡^

^‡^Mann-Whitney U test, for variables with non-normal distributions.

At 1-year follow-up, there was no significant difference of BMD change between clozapine and non-clozapine groups (p = 0.81, Mann-Whitney U test) (Table 2).

At 3-year follow-up, patients taking non-clozapine antipsychotics had more BMD loss than those in the clozapine group and healthy controls (p = 0.032 and 0.033, respectively, Mann-Whitney U test). There was no significant difference of BMD change between clozapine group and healthy controls (p = 0.33, Mann-Whitney U test) (Table 2).

For further examining the effects of PR and non-clozapine PS antipsychotics on BMD, we also classified patients into three groups based on their antipsychotics used: PR, non-clozapine PS, and clozapine. Overall, patients in PR and non-clozapine PS groups significantly lost BMD compared to clozapine-treated patients and healthy controls after 1–3 years (p = 0.037, Kruskal-Wallis test) (Supplementary Table 1).

### Predictive factors of bone density change in patients with schizophrenia

Backward multiple linear regressions were applied to identify the factors associated with BMD change (DEXA Z score difference after 1–3 years) in patients with schizophrenia (Supplementary Table 2). Potential factors included gender, education duration, age at onset, baseline BMD, duration of schizophrenia, duration of antipsychotic treatment, body weight, height, BMI, waist and hip circumstances, clozapine or non-clozapine antipsychotics use, PR or PS antipsychotics dose, concomitant mood stabilizers, PANSS scores, GAF score, calcium level, alkaline phosphatase level, TSH level, T3 level, cortisol level, estradiol level, testosterone level, prolactin level, and hyperprolactinemia.

Simple linear regressions separately examined all potentially variables aforementioned prior to application of multiple linear regressions. The variables with significant influence on the DEXA Z score change in single linear regressions were then selected to be variables for the multiple linear regression models.

For all patients with schizophrenia (including 1-year and 3-year follow-up patients), gender (B = −0.269, p = 0.033), age at onset (B = −0.017, p = 0.026), baseline DEXA Z score (B = −0.170, p < 0.001), serum calcium level (B = −0.378, p = 0.031) and T3 level (B = −0.005, p = 0.021) were associated with DEXA Z score change in simple linear regressions. In multiple linear regressions, only baseline DEXA Z score (B = −0.152, p = 0.001) and serum calcium level (B = −0.396, p = 0.019) were associated with DEXA Z score change (adjusted R square = 0.157, Supplementary Table 2). For schizophrenic patients with 3-year follow-up (N = 35), clozapine or non-clozapine antipsychotics use (B = 0.468, p = 0.080) were marginally associated with DEXA Z score change in simple linear regression.

For patients taking clozapine, serum calcium level (B = −0.713, p = 0.012) and T3 level (B = −0.008, p = 0.004) were associated with DEXA Z score change in simple linear regressions. In multiple linear regressions, serum calcium level (B = −0.607, p = 0.021) and T3 level (B = −0.077, p = 0.007) were also associated with DEXA Z score change (adjusted R square = 0.258, Supplementary Table 3).

## Discussion

It is important to monitor BMD change for schizophrenia patients with long-term, usually lifelong antipsychotics use. To our knowledge, the current study is the first one to follow up BMD after long-term use of clozapine versus non-clozapine (including PR and non-clozapine PS) antipsychotics in patients with chronic schizophrenia. The 3-year BMD change of healthy individuals was also measured for comparison. The findings suggest that patients receiving clozapine gained BMD, while those receiving non-clozapine (including PR and non-clozapine PS) antipsychotics lost BMD after 1–3 years. In consistent to our previous study that prolactin level itself was not correlated with BMD^12^, there was no significant association between prolactin level/hyperprolactinemia and BMD changes in this study. This longitudinal study conducted in a new cohort echoes the finding of our previous cross-sectional study in another cohort, suggesting that clozapine may have protective effect on BMD that may be unrelated to its prolactin-sparing effect^12^.

In the 1-year follow-up site, there was no significant difference of BMD change between clozapine and non-clozapine group. However, the baseline and endpoint BMD of the clozapine group were significantly higher than those of the non-clozapine group in the 1-year follow-up site. In the 3-year follow-up site, patients in the clozapine group had increased BMD, while patients in the non-clozapine group had decreased BMD after 3 years (Table 2). The insignificant difference of BMD change between clozapine and non-clozapine groups in the 1-year follow-up site may have been partially due to the relatively shorter duration of follow-up. Overall, our findings suggest that clozapine may be beneficial in protecting BMD among patients with schizophrenia after longer duration of follow-up.

Although the multiple linear regressions analysis did not show that clozapine or non-clozapine antipsychotics use was a significant predictive factor for BMD change in schizophrenic patients, it is interesting to elucidate clozapine’s effects on BMD.

The multiple linear regression models showed that serum calcium levels and T3 levels were negatively associated with BMD changes in patients receiving clozapine (Supplementary Table 3). A rat study found that clozapine can up-regulate calcium sensors, such as visinin-like protein 1 and neurocalcin δ^25^. An *in vitro* study also revealed that clozapine exerted activity by altering cell excitability and firing via actions on T-type calcium channels^26^. Thyroid function may be important for BMD in clozapine recipients. Studies have suggested the association between TSH level and BMD or fracture in both women and men^27–29^. Our previous study also found that TSH level was associated with BMD in women receiving clozapine^12^. However, studies that examine the interactions between clozapine and calcium or T3 are scanty. The role of calcium and T3 in BMD of clozapine recipients needs further investigation.

As aforementioned, clozapine may be able to protect BMD by activating NMDARs; however, other mechanisms deserve attention too. A recent animal study showed that clozapine had protective effect on bones possibly via causing sex-specific increase in pro-inflammatory cytokines^30^. It will be interesting to explore the effect of clozapine on bone mass and other related parameters.

This study has several limitations. First, the sample size was modest. Among the 35 schizophrenic patients with 3-year follow-up, only 13 patients were in the clozapine group. The insignificant finding for clozapine or non-clozapine antipsychotics use in the linear regression analysis may be partly due to the limited sample size and the relatively short follow-up duration (1 year) for some patients. Second, the follow-up durations and the exercise programs for the participants were different between the two hospitals. Third, it was difficult to restrict diet and exercise rigorously for patients and healthy controls at outpatient clinics although adequate education had been provided for them. Fourth, the amount of exercise was not measured quantitatively. Daily habits such as dietary calcium consumption^31,32^, exercise^9,33^ and sun exposure^9^ affect bone density. These factors should be included as variables in future study. Fifth, the bone metabolism related parameters were not measured completely due to the limited amount of blood sample. Sixth, measurements on prolactin, T3, calcium levels and several factors that may be associated with BMD at the endpoint were lacking, making it difficult to link baseline prolactin, T3, and calcium levels with BMD after 1- or 3-years follow up. Measurements on these factors should be included in future study. Lastly, the findings of the current study may not be unable to be extrapolated to all patients with schizophrenia because the population of the present study was in single race (Han Chinese) and had relatively long duration of disease and chronically ill symptoms.

In summary, this multicenter, longitudinal follow up study suggests that long-term clozapine treatment may be protective for BMD compared to non-clozapine antipsychotics in patients with chronic schizophrenia. The underlying mechanisms of clozapine’s effects on bone metabolism warrant further elucidation. Patients receiving long-term non-clozapine antipsychotic treatment are at higher risks for osteoporosis, fall and fracture. If the finding of this study can be replicated and confirmed in future studies, clozapine may be a potential choice for patients with schizophrenia who have high risk of osteoporosis. It is also interesting to demonstrate in prospective study whether switching from non-clozapine antipsychotics to clozapine helps to rescue BMD loss.

## Methods

### Setting

Patients with schizophrenia were recruited from outpatient clinics and chronic inpatient units of two major psychiatric centers in Taiwan (Changhua Hospital in central Taiwan [site 1] and Kaohsiung Chang Gung Memorial Hospital in southern Taiwan [site 2]). Healthy individuals including caregivers of patients were recruited from Kaohsiung Chang Gung Memorial Hospital. The study was approved by the institutional review boards of both hospitals (Institutional Review Board of Kaohsiung Chang Gung Memorial Hospital and Institutional Review Board of Changhua Hospital). All participants gave written informed consent in accordance with the Declaration of Helsinki after complete description of the study.

Both hospitals provided balanced diets which contained calcium about 600 mg and 2,000 calories per day for inpatients. Kaohsiung Chang Gung Memorial Hospital also provided regular daily exercise program (walking for one hour per day) for chronic inpatients.

### Participants

Patients with chronic schizophrenia were diagnosed and assessed by research psychiatrists using the DSM-IV criteria^34^. All patients had been under stable clinical condition with unchanged antipsychotics and doses for at least 6 months prior to the enrollment^35,36^. Patients recruited from Changhua Hospital were followed up for one year, while patients and healthy individuals recruited from Kaohsiung Chang Gung Memorial Hospital were followed up for three years. The antipsychotics and doses of patients from both sites had been kept unchanged during the follow-up period.

The patients were classified into two groups: clozapine group (clozapine was the only antipsychotic drug for the patients) and non-clozapine antipsychotics group. The non-clozapine antipsychotics included both PR (risperidone, amisulpride, paliperidone, ziprasidone or first-generation antipsychotics) and PS (olanzapine, quetiapine or aripiprazole) antipsychotics except for clozapine.

Healthy individuals were free from any axis I psychiatric disorder. Patients and healthy individuals with the following mental or physical conditions that might affect the BMD were excluded: substance abuse or dependence (including smoking and alcohol drinking that were forbidden at public places and hospitals in Taiwan), eating disorder, pregnancy or lactation, bone metabolism diseases, electrolyte imbalance, renal function impairment, pituitary tumor, thyroid or parathyroid diseases, and co-medications known to affect BMD (e.g. drugs for osteoporosis such as alendronate^37^, parathyroid hormone^38^, estrogens, selective estrogen receptor modulators^39^, bisphosphonates, and calcitonin^40^, heparin^41^, and glucocorticoids^35^) except antidepressants and benzodiazepines that did not consistently influence BMD^42^.

### Assessment

The BMD was measured by dual-energy X-ray absorptiometer (DEXA)^43–45^ at L2-L4 lumbar spine in a supine position at baseline and after 1-year (for site 1) or 3-year (for site 2) follow-up. The DEXA data was reviewed by experienced radiologists who were unaware of the clinical characteristics of the participants. Osteopenia was defined by an absolute DEXA T score between –2.5 and –1^46^, while osteoporosis was defined by a DEXA T score of –2.5 or lower^47^. T score ≤ –1 was defined as low BMD (LBMDT, including osteoporosis and osteopenia)^46^. T scores were obtained from the comparison with 30-year-old population. Therefore, we also measured DEXA Z score to help determine whether the bone density loss resulted from aging^48^. Z score ≤ –1 (LBMDZ) was considered as bone density loss due to causes other than age itself^48^.

Blood samples were collected at 8 AM. Bone-remodeling related factors were measured, including serum calcium, alkaline phosphatase, TSH (thyroid-stimulating hormone), T3, cortisol, estradiol, testosterone and prolactin. Complete blood and platelet count, BUN (blood urea nitrogen), creatinine, GOT (glutamic oxaloacetic transaminase) and GPT (glutamic pyruvic transaminase) were also measured to exclude major physical problems. Hyperprolactinemia is defined as an elevation of prolactin level above the upper limit of the reference laboratory, 25 ng/ml in women^49,50^ and 17 ng/ml in men^51^. Physical examinations including height, weight, body mass index (BMI), waist and hip circumstances were also determined.

For patients with schizophrenia, clinical data including psychiatric and physical illness history, medication kind and duration of use were collected by research psychiatrists by structured clinical interview and chart review. Positive and Negative Syndrome Scale (PANSS)^52,53^ was used to assess the severity of psychopathological symptoms. Global Assessment of Functioning (GAF) (axis V of DSM-IV) was also used to evaluate the overall functioning. The intra-class correlation coefficient (ICC) among the raters was 0.9.

### Statistical Analysis

Chi square tests or Fisher’s exact test was applied for the comparisons of categorical data between groups. Shapiro-Wilk W test was used to examine the normality. Continuous data were analyzed by t-test and ANOVA test, or the corresponding non-parametric method, Mann-Whitney U test and Kruskal-Wallis test, for variables with non-normal distributions. The multiple regression models were applied to explore the contributing factors related to DEXA Z score change after 1–3 years. All tests were two-tailed, and significance of tests was defined as p-value < 0.05. Data were analyzed with SPSS version 18.0 (SPSS Inc., Chicago, IL, USA).

### Role of the Sponsor

The sponsors were not involved in the design and conduct of the study; collection, management, analysis, and interpretation of the data; and preparation, review, or approval of the manuscript.

## Supplementary information

Supplementary tables
