# Supplementary material for: Long-term Use of Clozapine is Protective for Bone Density in Patients with Schizophrenia
Source: Sci Rep. 2019 Mar 7;9:3895. doi: 10.1038/s41598-019-40691-7 (PMC6405997; doi:10.1038/s41598-019-40691-7)
Supplement: Supplementary file 1 — Supplementary tables [file 41598_2019_40691_MOESM1_ESM.doc]

Abstract: 248 words; Text: 2915 words; 53 references; 2 Tables; 3 Supplementary Tables

Long-term Use of Clozapine is Protective for Bone Density in Patients with Schizophrenia

Chieh-Hsin Lin, M.D., Ph.D. ^a, b, c^, Chun-Yuan Lin, M.D., Ph.D. ^d, e, f^, Hong-Song Wang, M.D., MSc ^g^, Hsien-Yuan Lane, M.D., Ph.D. ^b, h, i, *^

^a^ Department of Psychiatry, Kaohsiung Chang Gung Memorial Hospital, Chang Gung University College of Medicine, Kaohsiung, Taiwan

^b^ Graduate Institute of Biomedical Sciences, China Medical University, Taichung, Taiwan

^c^ School of Medicine, Chang Gung University, Taoyuan, Taiwan

^d^ Tsaotun Psychiatric Center, Ministry of Health and Welfare, Nantou, Taiwan

^e^ School of Medicine, Chung Shan Medical University, Taichung, Taiwan

^f^ National Changhua University of Education, Changhua, Taiwan

^g^ Psychiatric department, Changhua Hospital, Ministry of Health & Welfare, Changhua, Taiwan

^h^ Department of Psychiatry & Brain Disease Research Center, China Medical University Hospital, Taichung, Taiwan

^i^ Department of Psychology, College of Medical and Health Sciences, Asia University, Taichung, Taiwan

Running Title: Long-term clozapine use protects BMD in schizophrenia

*Reprints: Hsien-Yuan Lane, M.D., Ph.D, Department of Psychiatry, China Medical University Hospital, No. 2, Yuh-Der Road, Taichung 404, Taiwan (email address: hylane@gmail.com).

| **Supplementary Table 1**. Bone mineral density change among patients with schizophrenia (on PR, non-clozapine PS or clozapine antipsychotics) and healthy individuals | | | | | | |
| --- | --- | --- | --- | --- | --- | --- |
|  | Controls | PR antipsychotics | Non-clozapine PS antipsychotics | Clozapine | P value | |
| **DEXA Z score, overall** | (N=44) | (N=47) | (N=22) | (N=42) |  | |
| Baseline, mean (SD) | 0.28 (1.09) | -0.49 (1.17) | 0.16 (0.94) | 0.38 (1.45) | 0.001 | |
| Endpoint, mean (SD) | 0.42 (1.08) | -0.64 (1.00) | -0.03 (0.72) | 0.41 (1.49) | <0.001^‡^ | |
| Difference, mean (SD) | 0.16 (0.47) | -0.14 (0.69) | -0.20 (0.62) | 0.03 (0.64) | 0.037^‡^ | |
| **DEXA Z score, 1 year follow up** |  | (N=35) | (N=13) | (N=29) |  | |
| Baseline, mean (SD) | -- | -0.73 (1.02) | -0.02 (1.00) | 0.16 (1.49) | 0.012 | |
| Endpoint, mean (SD) | -- | -0.84 (0.93) | -0.24 (0.53) | 0.09 (1.47) | 0.006 | |
| Difference, mean (SD) | -- | -0.10 (0.49) | -0.22 (0.74) | -0.08 (0.70) | 0.800^‡^ | |
| **DEXA Z score, 3 year follow up** | (N=44) | (N=12) | (N=9) | (N=13) |  | |
| Baseline, mean (SD) | 0.28 (1.09) | 0.17 (1.35) | 0.43 (0.82) | 0.88 (1.29) | 0.359 | |
| Endpoint, mean (SD) | 0.42 (1.08) | -0.05 (0.98) | 0.27 (0.88) | 1.13 (1.29) | 0.095^‡^ | |
| Difference, mean (SD) | 0.16 (0.47) | -0.25 (1.11) | -0.17 (0.43) | 0.25 (0.41) | 0.089^‡^ | |
| Abbreviations: PR, prolactin-raising; PS, prolactin-sparing  ^‡^ Kruskal-Wallis test, for variables with non-normal distributions | | | | | |  |

Abbreviations: PR, prolactin-raising; PS, prolactin-sparing; PANSS: Positive and Negative Syndrome Scale.

***Prior to application of multiple linear regressions, simple linear regression models separately examined all potentially predicting variables. All the variables which influenced the DEXA Z score change in single linear regressions were then selected to be predicting variables for the multiple linear regression models.**

| **Supplementary Table 2***.* Simple and multiple linear regression analyses of independent predictive factors* associated with DEXA Z score change in patients with schizophrenia (backward) | | | | | |
| --- | --- | --- | --- | --- | --- |
|  | Univariate | | Multivariate | | |
| Variables | B (SE) | *P* | B (SE) | t | *P* |
| Gender | -0.269 (0.125) | **0.033** |  |  |  |
| Education duration (y) | 0.020 (0.021) | 0.357 |  |  |  |
| Age at onset (y) | -0.017 (0.007) | **0.026** |  |  |  |
| Baseline DEXA Z score | -0.170 (0.045) | **<0.001** | -0.152 (0.046) | -3.289 | **0.001** |
| Duration of disease (month) | 0.001 (0.001) | 0.203 |  |  |  |
| Duration of antipsychotic treatment (day) | 0.000 (0.000) | 0.102 |  |  |  |
| Body weight (Kg) | 0.000 (0.005) | 0.949 |  |  |  |
| Height (cm) | 0.007 (0.008) | 0.349 |  |  |  |
| Body mass index (BMI) | -0.007 (0.015) | 0.658 |  |  |  |
| Waist circumstance (cm) | -0.012 (0.006) | 0.055 |  |  |  |
| Hip circumstance (cm) | -0.012 (0.007) | 0.091 |  |  |  |
| Clozapine or non-clozapine antipsychotics use | 0.184 (0.128) | 0.153 |  |  |  |
| PR antipsychotics dose (mg/day) | 0.000 (0.000) | 0.563 |  |  |  |
| PS antipsychotics dose (mg/day) | 0.000 (0.000) | 0.909 |  |  |  |
| Concomitant mood stabilizers | 0.019 (0.157) | 0.902 |  |  |  |
| PANSS positive-subscale score | 0.000 (0.014) | 0.983 |  |  |  |
| PANSS negative-subscale score | 0.007 (0.016) | 0.672 |  |  |  |
| PANSS general-subscale score | -0.005 (0.008) | 0.507 |  |  |  |
| Global Assessment of Functioning score | -0.002 (0.006) | 0.765 |  |  |  |
| Serum calcium (mg/dl) | -0.378 (0.173) | **0.031** | -0.396 (0.167) | -2.376 | **0.019** |
| Alkaline phosphatase (U/L) | 0.006 (0.003) | 0.066 |  |  |  |
| TSH (mIU/L) | 0.005 (0.010) | 0.596 |  |  |  |
| T3 (ng/dl) | -0.005 (0.002) | **0.021** |  |  |  |
| Cortisol (μg/dl) | 0.009 (0.015) | 0.564 |  |  |  |
| Estradiol (pg/ml) | -0.002 (0.002) | 0.314 |  |  |  |
| Testosterone (pg/ml) | 0.020 (0.024) | 0.415 |  |  |  |
| Prolactin (ng/ml) | 0.000 (0.003) | 0.963 |  |  |  |
| Hyperprolactinemia | 0.087 (0.128) | 0.499 |  |  |  |
| Adjusted R square = 0.157 |  |  |  |  |  |

| **Supplementary Table 3***.* Simple and multiple linear regression analyses of independent predictive factors* associated with DEXA Z score change in schizophrenia patients receiving clozapine (backward) | | | | | |
| --- | --- | --- | --- | --- | --- |
|  | Univariate | | Multivariate | | |
| Variables | B (SE) | *P* | B (SE) | t | *P* |
| Gender | -0.271 (0.198) | 0.178 |  |  |  |
| Education duration (y) | -0.014 (0.035) | 0.683 |  |  |  |
| Age at onset (y) | 0.011 (0.019) | 0.565 |  |  |  |
| Baseline DEXA Z score | -0.073 (0.068) | 0.289 |  |  |  |
| Duration of disease (month) | 0.000 (0.001) | 0.655 |  |  |  |
| Duration of antipsychotic treatment (day) | 0.000 (0.000) | 0.227 |  |  |  |
| Body weight (Kg) | -0.001 (0.009) | 0.909 |  |  |  |
| Height (cm) | -0.006 (0.013) | 0.612 |  |  |  |
| Body mass index | 0.007 (0.026) | 0.780 |  |  |  |
| Waist circumstance (cm) | -0.012 (0.011) | 0.265 |  |  |  |
| Hip circumstance (cm) | -0.017 (0.012) | 0.164 |  |  |  |
| Clozapine dose (mg/day) | 0.000 (0.001) | 0.572 |  |  |  |
| Concomitant mood stabilizers | 0.165 (0.241) | 0.498 |  |  |  |
| PANSS positive-subscale score | 0.006 (0.025) | 0.810 |  |  |  |
| PANSS negative-subscale score | 0.012 (0.023) | 0.609 |  |  |  |
| PANSS general-subscale score | 0.015 (0.012) | 0.214 |  |  |  |
| Global Assessment of Functioning score | -0.004 (0.010) | 0.661 |  |  |  |
| Serum calcium (mg/dl) | -0.713 (0.272) | **0.012** | -0.607 (0.253) | -2.400 | **0.021** |
| Alkaline phosphatase (U/L) | 0.004 (0.004) | 0.306 |  |  |  |
| TSH (mIU/L) | 0.004 (0.010) | 0.647 |  |  |  |
| T3 (ng/dl) | -0.008 (0.003) | **0.004** | -0.077 (0.002) | -2.857 | **0.007** |
| Cortisol (μg/dl) | 0.013 (0.023) | 0.571 |  |  |  |
| Estradiol (pg/ml) | -0.001 (0.003) | 0.783 |  |  |  |
| Testosterone (pg/ml) | 0.070 (0.046) | 0.133 |  |  |  |
| Prolactin (ng/ml) | 0.001 (0.013) | 0.910 |  |  |  |
| Hyperprolactinemia | 0.358 (0.334) | 0.290 |  |  |  |
| Adjusted R square = 0.258 |  |  |  |  |  |

Abbreviation: PANSS: Positive and Negative Syndrome Scale.

*Prior to application of multiple linear regressions, simple linear regression models separately examined all potentially predicting variables. All the variables which influenced the DEXA Z score change in single linear regressions were then selected to be predicting variables for the multiple linear regression models.
